# Effectiveness of front-of-pack nutrition labels in Saudi Arabia: a comparative study

**DOI:** 10.3389/fnut.2026.1811411

**Published:** 2026-05-25

**Authors:** Nahla Mohammed Bawazeer, Abeer Salman Alzaben, Shahd Alabdulkader, Fatmah Almoayad, Abeer A. Aljahdali, Mona Alatawi, Ohood Mahdi Hussain Mobaraki, Fatmah Abdullah Alrayes, Nada Benajiba

**Affiliations:** 1 Department of Health Sciences, College of Health and Rehabilitation Sciences, Princess Nourah Bint Abdulrahman University, Riyadh, Saudi Arabia; 2 Department of Clinical Nutrition, Faculty of Applied Medical Sciences, King Abdulaziz University, Jeddah, Saudi Arabia; 3 Department of Nutritional Sciences, University of Michigan, Ann Arbor, MI, United States; 4 Department of Clinical Nutrition, King Khalid Hospital, Tabuk, Saudi Arabia; 5 Department of Clinical Nutrition, Medical City, Jazan University, Jazan, Saudi Arabia; 6 Food Service Department, Eastern Health Cluster, Dammam, Saudi Arabia; 7 Unité Mixte de Recherche en Nutrition et Alimentation URAC 39 (Université Ibn Tofaïl–CNESTEN), RDC-Nutrition, Kenitra, Morocco

**Keywords:** food labelling, FoPL, front-of-pack nutrition label, Nutri-Score, Saudi Arabia

## Abstract

**Objectives:**

To evaluate Saudi consumers’ understanding and food choices based on five FoPL systems using a validated methodology adapted from the international FOP-ICE (Front-Of-Pack International Comparative Experimental) study.

**Methods:**

An online survey was administered to 1,500 Saudi adults (≥18 years) from all five regions. Eligible participants regularly purchased yoghurt, biscuits, or breakfast cereals. Participants were randomly assigned to view products with or without FoPLs and completed a structured questionnaire assessing their food choices and label understanding.

**Results:**

Among respondents, 56.9% were female, and 47.4% were primarily responsible for grocery shopping. The greatest improvement in correct product choices was observed with the Multiple Traffic Light (MTL) label (31%), followed by Health Warnings (28%) and Nutri-Score (27%). The strongest associations with accurate nutritional ranking were observed for MTL (OR = 2.18, *p* < 0.001), followed by Health Warnings (OR = 1.83, *p* < 0.001) and Nutri-Score (OR = 1.56, *p* = 0.003). Nutri-Score and Health Star Rating (HSR) improved biscuit selection, whereas Health Warnings, HSR, and MTL influenced cereal choices (*p* < 0.05). Significant associations with changes in nutritional quality were observed for gender, place of residence, responsibility for grocery shopping, and self-reported nutrition knowledge.

**Conclusion:**

All FoPL systems improved food choices compared with no labelling. MTL, Health Warnings, and Nutri-Score were the most effective and well-received. MTL showed strong potential to guide healthier choices, whereas Nutri-Score may gain impact through broader public education and awareness.

## 1 Introduction

According to the World Health Organization (WHO), non-communicable diseases accounted for 73% of all deaths in Saudi Arabia (1). In addition, micronutrient deficiencies—particularly Vitamin D and iron—are highly prevalent among Saudi adults (2, 3). In response to this situation, the Saudi Food and Drug Authority implemented policies and regulations to combat nutrition-related health problems, including mandatory labelling of pre-packaged foods since 2013 (4). These labelling regulations require the disclosure of key nutritional information, including the amounts of calories, carbohydrates, proteins, and fats (4).

Food labels are a recognized tool for raising awareness about eating behaviors, food quality, and daily nutrient needs, as they provide consumers with dietary guidelines (5). According to Lewis et al. (6), individuals with chronic diseases were more likely to check and use nutrition labels and demonstrated greater awareness of national dietary recommendations than those without such conditions. This indicates that the use of food labels could contribute to the prevention of diet-related health issues. However, Washi (7) found that most Saudi consumers lacked knowledge of nutritional content, serving sizes, and health claims presented on food labels. Similarly, Al-Barqi et al. (8) reported that only 27.4% of female students “always” or “usually” used food labels when purchasing food, highlighting the need for adequate nutrition knowledge to facilitate effective label use. A recent review by Benajiba et al. (9) concluded that nutritional labels, as a population-based intervention, could positively affect individual behavior and enhance public health in Arab countries. However, the effectiveness of this strategy largely depends on consumers’ ability to understand and effectively use the information on nutrition labels. Consequently, the impact of food labelling remains under debate, as it can only be demonstrated if labels are used properly and consistently by consumers. Therefore, a new format for nutrition labelling is highly recommended.

**Figure 1:**
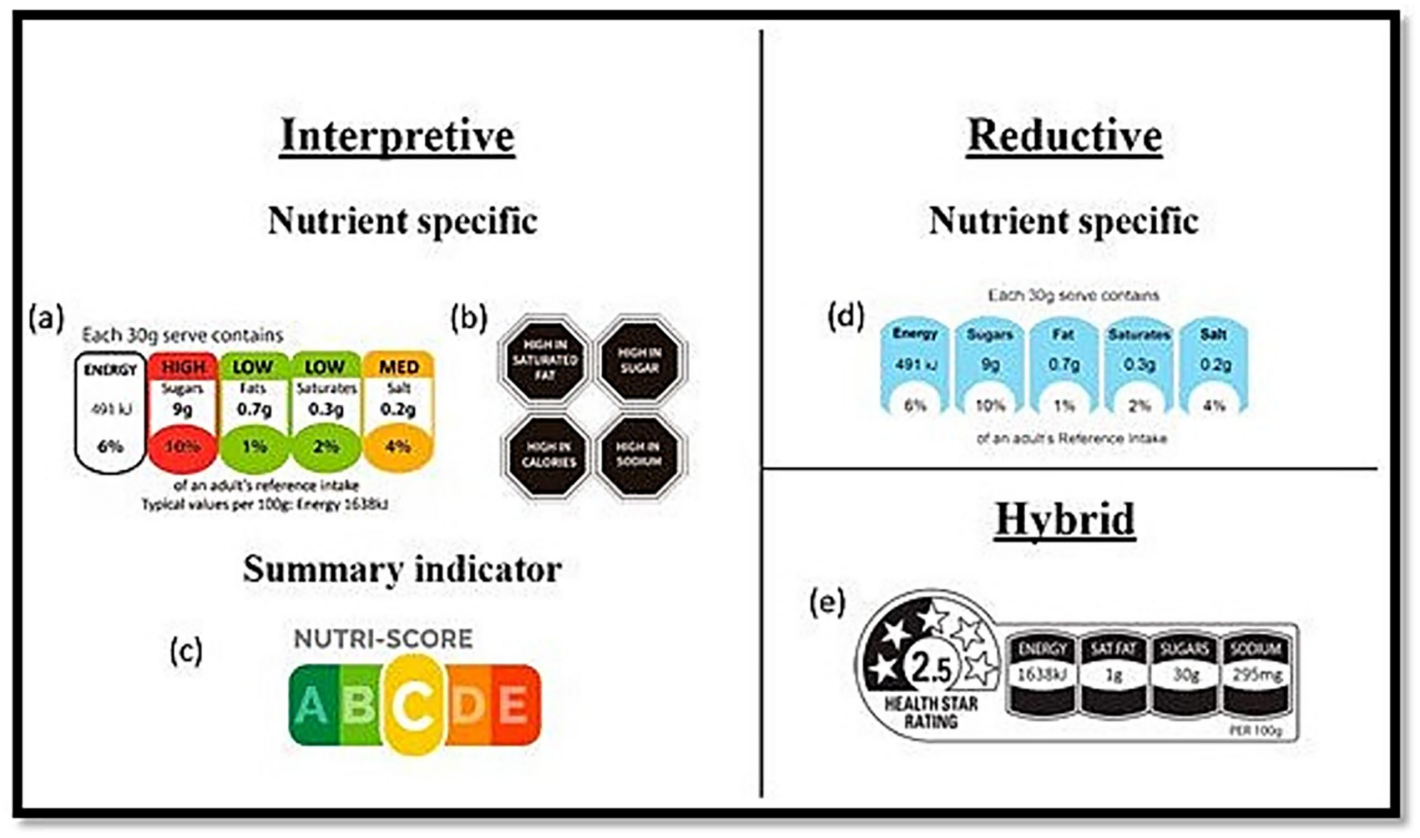
The five studied nutrition labels: **(a)** Multiple Traffic Light (MTL), **(b)** Warnings **(c)** Nutri-Score, **(d)** Reference Intakes, and **(e)** Health Star Rating. Adapted from (18), licensed under CC BY 4.0.

In 2017, Julia and Hercberg (10) developed the Nutri-Score, a front-of-pack nutrition label (FoPL) with a validated algorithm and graphical format grounded in scientific evidence. Despite its growing adoption, the suitability and effectiveness of the Nutri-Score graphical format within the Saudi context have yet to be evaluated. Thus, it is important to evaluate the ability of five FoPL systems, including the Health Star Rating System (HSR), Multiple Traffic Lights (MTLs), Reference Intakes (RIs), Warning symbols, and Nutri-score (Figure 1), to assist consumers in assessing the nutritional quality of various foods and making informed choices (11). A study conducted across 12 countries identified Nutri-Score as the most effective FoPL for enabling consumers to compare the nutritional quality of products. Its superior discriminative capacity based on nutritional composition was consistent across countries and aligned well with existing nutritional recommendations (12, 13).

In this study, we examined Saudi consumers’ food choices and their objective understanding of five different FoPL systems using an online experimental questionnaire, adapted from the validated Front-Of-Pack International Comparative Experimental (FOP-ICE) framework (11, 14). We hypothesised that interpretive FoPL systems would result in significantly greater objective understanding of nutritional quality and healthier food choices than non-interpretive or numeric labelling formats, such as RIs, among Saudi adults. This hypothesis was tested by experimentally comparing consumers’ comprehension and food selection responses across five FoPL formats using a standardised and previously validated methodology. By extending the existing evidence from Saudi Arabia through a multi-label comparative approach (14) and objective understanding in diverse populations (15, 16), this study aims to advance the field by strengthening the empirical basis for FoPL policy development and supporting the identification of more effective labelling strategies to encourage healthier dietary behaviors in Saudi populations.

## 2 Materials and methods

### 2.1 Study design

An online cross-sectional survey was administered between September 2022 and December 2023, following the methodology established in the international FOP-ICE study framework (11). The study protocol was further adapted to the Saudi Arabian context, with modifications to the data collection procedures and selection of food products for testing. Detailed procedural steps are described in sections 2.4.1–2.4.3.

### 2.2 Population and sampling

**Figure 2:**
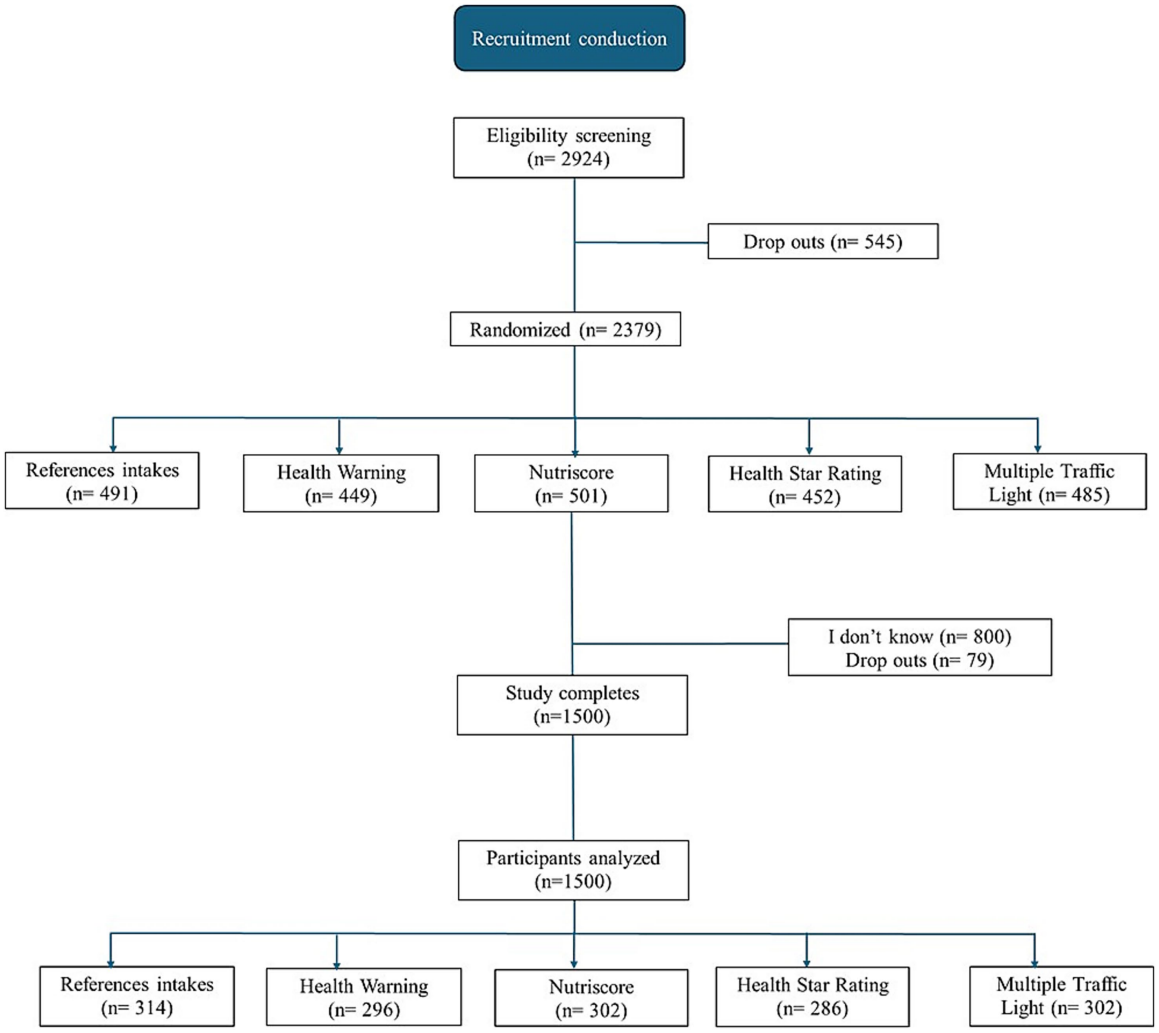
Study sample flow diagram.

Participants were randomised to one of five FoPLs using SurveyMonkey’s built-in randomisation function (allocation ratio 1:1:1:1:1). A total of 2,924 participants took part in this study. Of these, 545 dropped out and did not complete the questionnaire. Of the remaining 2,379 participants, 800 were excluded because they reported not knowing how to read the label, and 79 did not complete the questionnaire. Consequently, 1,500 participants were included in the analysis (Figure 2).

Participants were selected through quota sampling based on sex, age, household income, and geographic region. Quotas were monitored during data collection to ensure balanced recruitment across categories. Sex representation was nearly equal (50% males and 50% females). Age distribution was stratified as follows: one-third of the participants were under 28 years of age; one-third were between 28 and 39 years; and one-third were 40 years or older. Household income was categorised based on national statistics as follows: low (<7,750 SAR/month), medium (7,750–15,300 SAR/month), and high income (>15,300 SAR/month). These income brackets were calculated around the national median household income as reported in 2018 by the General Authority of Statistics in Saudi Arabia (17). The middle-income group was defined as within ±33% of the median income, with incomes below or above this range classified as low or high, respectively (18). Additionally, one-fifth of the study population was sampled from each of the five regions of Saudi Arabia (North, South, Centre, East, and West) (19).

Eligibility criteria included being a regular purchaser of at least one of the following food types: yoghurt, biscuits, or breakfast cereals, defined as an individual with no restrictions on purchasing these products.

### 2.3 Ethical considerations

The study was approved by the Institutional Review Board of Princess Nourah Bint Abdulrahman University (IRB 22-0410, 28 Aug 2022). Participation was voluntary, with electronic informed consent obtained, and confidentiality maintained. Data were collected via an online questionnaire distributed through social media, email, and professional mailing lists.

### 2.4 Survey instrument and experimental procedure

The questionnaire was administered in Arabic and pre-tested for validity and reliability, as described by Aguenaou et al. (20). All nutritional labels were tested equally, and randomisation was applied using the online SurveyMonkey platform. Each participant was exposed to only one FoPL condition throughout the study.

#### 2.4.1 Nutritional labels tested

Five FoPLs were evaluated:

RIs: Used by food manufacturers in several countries since 2006.

Warning Labels: Symbol-based health warnings, first introduced in Chile in 2016.

Nutri-Score: Introduced in France in 2017 and later adopted by other European countries, including Spain, Belgium, the Netherlands, Germany, Luxembourg, and Switzerland.

HSR: A rating system in use across Australia and New Zealand since 2014.

MTLs: A colour-coded label system used in the United Kingdom since 2005.

#### 2.4.2 Choice of products

**Figure 3:**
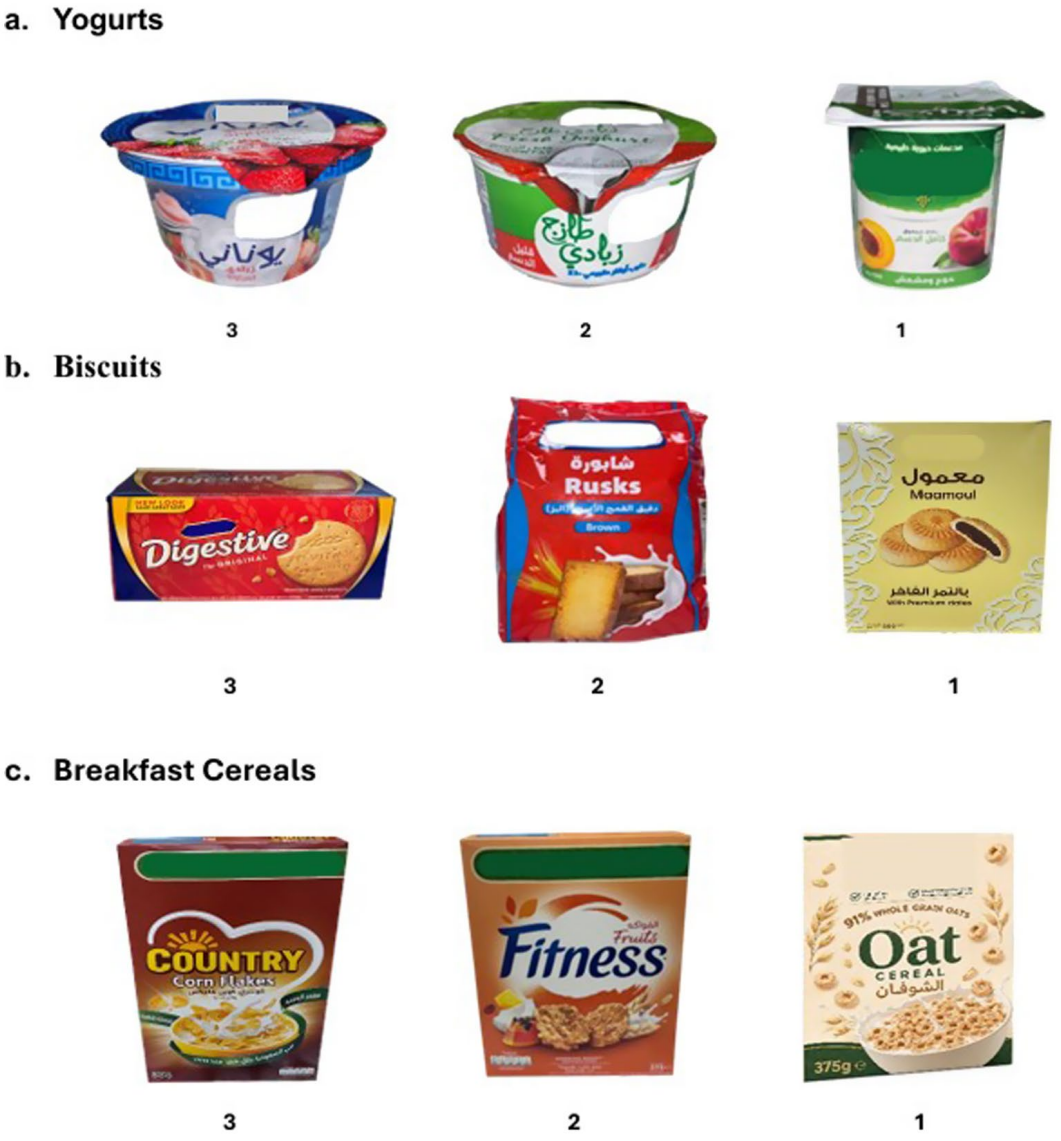
Classification of food products by nutritional quality: (a) Yogurt, (b) Biscuits, and (c) Breakfast cereals.

Three product categories were selected: yoghurt, biscuits, and breakfast cereals. These products were chosen based on three criteria: they are processed and commonly consumed by the Saudi population; they are well recognized; and their nutrient composition shows variability across products (Figure 3). For each category, products were classified into three levels of nutritional quality (low, moderate, and high) based on their nutrient content, including energy, total sugar, total fat, saturated fat, and sodium, allowing for comparative analysis for each category. Each product was evaluated against predefined criteria derived from the respective FoPL scheme and then categorized accordingly into low, moderate, or high nutritional quality (21). The labels were consistently placed on the packaging to prevent other nutritional details or quality indicators from affecting participants’ perceptions.

#### 2.4.3 Experimental procedure

The questionnaire was divided into two main sections. The first section collected demographic and background information, including sex, age, marital status, household income, level of education, occupation, participation in supermarket shopping, perceived diet quality, and self-assessed nutrition knowledge. Participants were also asked whether they had noticed the label during the survey.

The second section assessed participants’ food choices and their objective understanding of food labels. To minimise bias towards label awareness, the study was conducted in two stages using the SurveyMonkey platform: (a) evaluation of food choices, and (b) assessment of objective understanding.

Participants were first shown three sets of selected food products (yoghurt, biscuits, and breakfast cereals) without FoPLs. They were asked to imagine purchasing each type of product and to select their preferred option. Subsequently, they were instructed to rank the three products in each category based on perceived nutritional quality as follows: (1) best, (2) intermediate, and (3) worst. The option of “I don’t know” was also provided. These selection and ranking tasks were carried out independently for each food category.

The same tasks were repeated with FoPLs displayed on the products. The sequence of product categories was randomised for each respondent to reduce order effects.

### 2.5 Data analysis and statistics

Food choices were scored from 1 (lowest) to 3 (highest nutritional quality) for each product under no-label and FoPL conditions; “I wouldn’t buy any” received no score. Difference scores per category (FoPL minus no-label) ranged from −2 to 2 and were summed across three categories (−6 to +6). Proportions of improved or worsened choices were calculated by label type and category. Multivariate ordinal logistic regression examined associations between label type and scores. Objective understanding was assessed by correct ranking of all three products per category, with changes between conditions expressed as percentages.

The primary outcome measure was the difference in the number of correct responses between FoPL and no-label conditions. The score for each category ranged from −1 (deteriorated) to +1 (improved), with 0 indicating no change. All three category scores were then summed to produce a total global score ranging from −3 to +3. Participants who selected “I don’t know” and did not provide rankings were excluded from the analysis.

A multivariate ordinal logistic regression model was employed to investigate the association between food choice score and label type. Based on prior findings indicating lower performance of the RI label (11), it was designated as the reference category in the regression analyses. Covariates included age, sex, place of residence, marital status, education, occupation, household income, and grocery shopping responsibility. Statistical significance was set as *p* < 0.05. All statistical analyses were performed using the R Software (version 4.4.0; R Foundation for Statistical Computing, Vienna, Austria). Chi-square and Fisher–Freeman–Halton tests were used to assess the association between sociodemographic characteristics and changes in nutritional quality across food categories.

## 3 Results

### 3.1 Participants’ characteristics

Participants were randomly allocated to one of five FoPL types, resulting in a relatively even distribution across all label groups, with each comprising approximately 20% of the total sample (*N* = 1,500) (Table 1).

**Table 1:** Sample distribution across FoPL groups.

Type of FoPLs	Frequency *n*, percentage (%)
References Intakes (RI)	314 (20.93)
Health Warning	296 (19.73)
Nutri Score	302 (20.13)
Health Star Rating (HSR)	286 (19.07)
Multiple Traffic Light (MTL)	302 (20.13)

Data are presented as numbers *n* (%).

Table 2 presents the demographic characteristics of the 1,500 participants. The sample was relatively balanced in terms of age, with approximately one-third of participants in each of the three age groups: <28 years (33.3%), 28–40 years (34.6%), and >40 years (32.1%). Females accounted for 56.9% of participants, drawn from all five regions of Saudi Arabia, with the largest proportion from the Eastern region (26.2%). Most respondents were married (52.7%), held a diploma or university degree (64.2%), and were employed (55.5%). The majority reported a monthly household income between 7,750 and 15,300 SAR (approximately 2,066–4,080 USD). Nearly half of the participants (47.40%) reported being primarily responsible for grocery shopping.

**Table 2:** Demographic characteristics of the study participants (*N* = 1,500).

Variable	Frequency *n*, percentage (%)
Age (years)	
<28	500 (33.33)
28–40	519 (34.60)
≥40	481 (32.07)
Gender	
Male	647 (43.13)
Female	853 (56.87)
Place of residence (regions)	
Central	295 (19.67)
Eastern	393 (26.20)
Northern	271 (18.07)
Southern	236 (15.73)
Western	305 (20.33)
Marital status	
Unmarried (single, divorced, widowed)	710 (47.3)
Married	790 (52.7)
Educational level	
High school or less	248 (16.53)
Diploma/University	963 (64.20)
Postgraduate	289 (19.27)
Employment status	
Working	915 (61.0)
Not working & students	585 (39.0)
Monthly income level	
<7,750	455 (30.33)
7,750–15,300	563 (37.53)
>15,300	481 (32.07)
Responsible for grocery shopping	
Yes	711 (47.40)
No	365 (24.33)
All family members	423 (28.20)
Participants’ perception of their diet	
Very well balanced	137 (9.13)
Balanced	586 (39.07)
Somewhat balanced	622 (41.47)
Unbalanced	154 (10.27)
Nutritional knowledge	
Extensive knowledge of nutrition	552 (36.80)
Sufficient knowledge of nutrition	736 (49.07)
Limited knowledge of nutrition	180 (12.00)
No knowledge of nutrition	32 (2.13)
Frequency of buying yoghurt	
Always	882 (58.80)
Often	338 (22.53)
Sometimes	201 (13.40)
Rarely	63 (4.20)
Never	15 (1.00)
Frequency of buying biscuits	
Always	359 (23.93)
Often	379 (25.27)
Sometimes	550 (36.67)
Rarely	185 (12.33)
Never	20 (1.33)
Frequency of buying breakfast cereals	
Always	392 (26.13)
Often	289 (19.27)
Sometimes	419 (27.93)
Rarely	304 (20.27)
Never	90 (6.00)

Data are presented as numbers *n* (%).

Regarding dietary perception, most participants rated their diet as either slightly balanced (41.47%) or balanced (39.07%). Self-reported nutritional knowledge was high or adequate for 85.87% of participants. Regarding purchasing habits, yoghurt was the most frequently purchased item, with 58.80% of participants reporting that they “always” bought it. However, biscuits and breakfast cereals were more often purchased “sometimes” (36.67 and 27.93%, respectively), indicating moderate consumption patterns.

### 3.2 Food choices and objective understanding

**Figure 4:**
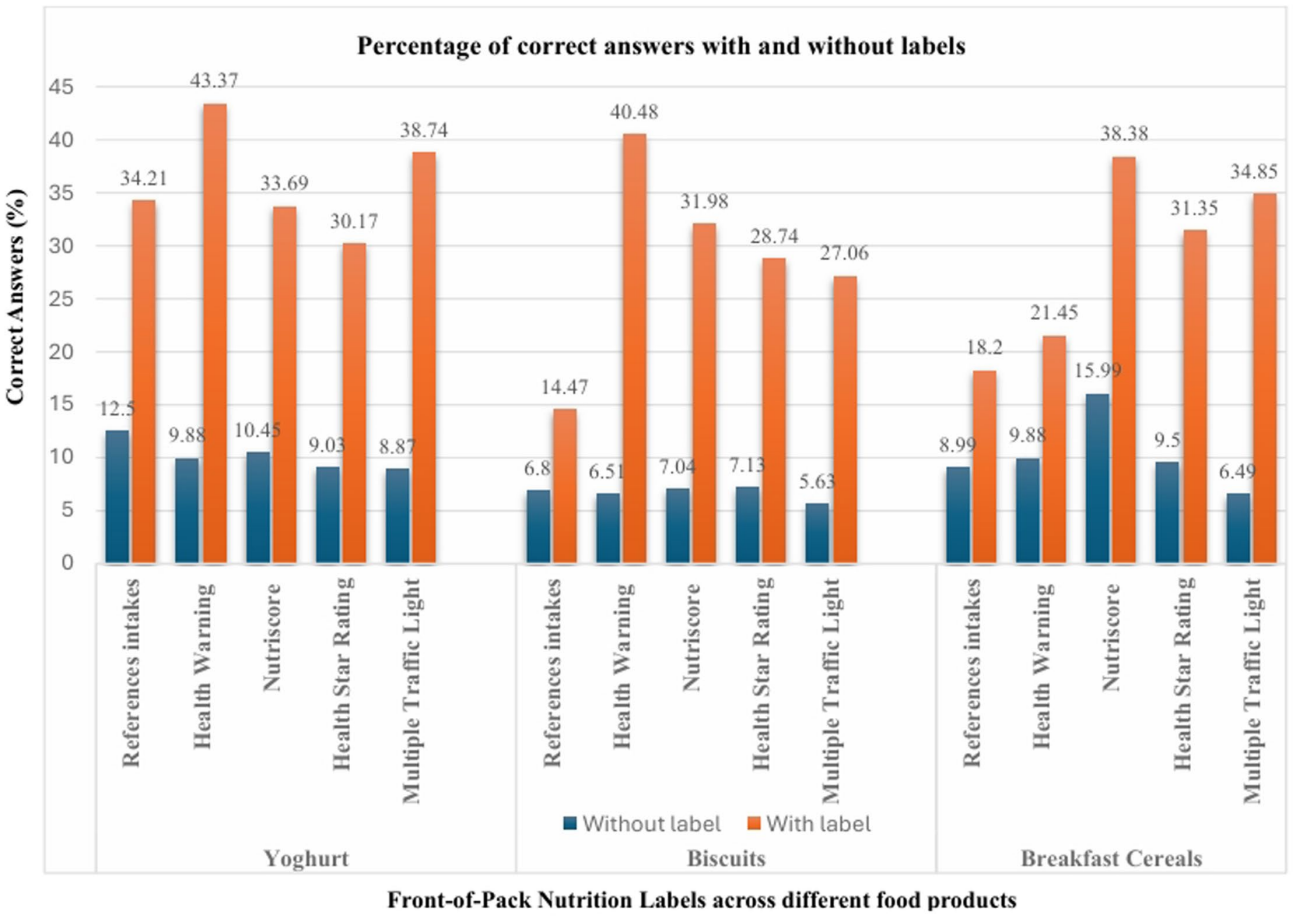
Correct ranking of products by nutritional quality with and without nutrition labels for yoghurt, biscuits, and breakfast cereals.

Figure 4 illustrates the percentage change in product choices for yoghurt, biscuits, and breakfast cereals following the introduction of various FoPLs, including RI, Health Warning, Nutri-Score, HSR, and MTL. Among all labels, MTL led to the greatest improvement in correct responses across all food categories, with an average improvement of 31% (46.03% vs. 10.93% for yoghurt; 33.11% vs. 7.28% for biscuits; and 41.72% vs. 8.28% for breakfast cereals). Health Warning labels improved responses by approximately 28% (46.96% vs. 11.49% for yoghurt; 45.08% vs. 6.76% for biscuits; and 24.32% vs. 11.82% for breakfast cereals), whereas the Nutri-Score label resulted in 27% improvement (39.74% vs. 13.25% for yoghurt; 37.75% vs. 8.94% for biscuits; and 47.02% vs. 20.86% for breakfast cereals). HSR showed a slightly lower effect, with an average improvement of approximately 24% (35.31% vs. 11.19% for yoghurt; 34.27% vs. 9.44% for biscuits; and 37.54% vs. 12.98% for breakfast cereals). In comparison, the RI label demonstrated the least improvement in correct responses (38.85% vs. 12.74% for yoghurt; 16.93% vs. 8.60% for biscuits; and 19.43% vs. 10.83% for breakfast cereals).

The findings of the ordinal logistic regression analysis are presented in Table 3. Compared with the RI label, most FoPL were not significantly associated with changes in the nutritional quality of food choices, either overall or by food category. However, significant improvements were identified for biscuits with the Nutri-Score [odds ratio (OR) = 1.36 (1.01–1.83), *p* = 0.04] and HSR labels [OR = 1.46 (1.08–1.98), *p* = 0.014]. For breakfast cereals, improvements were associated with Health Warning [OR = 0.56 (0.68–1.35), *p* < 0.001], HSR [OR = 0.61 (0.76–1.52), *p* = 0.002], and MTL labels [OR = 0.70 (0.96–1.90), *p* = 0.021], respectively.

**Table 3:** Relationship between FoPL type and change in nutritional quality of food choices across labels and food categories (*N* = 1,500).

Product choice	OR	95% CI	*p*-value
Yogurt			
Health Warning	0.96	0.68–1.35	0.813
Nutri-Score	1.36	0.97–1.92	0.075
HSR	1.08	0.76–1.52	0.675
MTL	1.35	0.96–1.90	0.088
Biscuits			
Health Warning	1.07	0.80–1.44	0.638
Nutri-Score	1.36	1.01–1.83	0.040*
HSR	1.46	1.08–1.98	0.014*
MTL	1.09	0.81–1.47	0.549
Breakfast cereals			
Health Warning	0.56	0.68–1.35	<0.001^*^
Nutri-Score	0.9	0.97–1.92	0.501
HSR	0.61	0.76–1.52	0.002^*^
MTL	0.7	0.96–1.90	0.021^*^
All products			
Health Warning	0.76	0.57–1.01	0.576
Nutri-Score	1.21	0.91–1.61	0.19
HSR	1.04	0.78–1.39	0.802
MTL	0.93	0.70–1.24	0.641

^*^The RI label served as the reference category in the multivariate ordinal logistic regression analyses. Models were adjusted for age, sex, place of residence, marital status, education level, occupation, household, and grocery shopping responsibility. OR: odds ratio; CI: confidence interval; ^*^p < 0.05.

Table 4 displays the associations between the different FoPLs and participants’ accuracy in ranking products by nutritional quality, using the RI label as the reference. MTL demonstrated the highest improvement in classification accuracy across all product categories [OR = 2.18 (1.63–2.91), *p* < 0.001], followed by Health Warning labels [OR = 1.83 (1.37–2.44), *p* < 0.001], Nutri-Score [OR = 1.56 (1.16–2.10), *p* = 0.003], and HSR [OR = 1.46 (1.78–1.98), *p* = 0.014]. The magnitude of these effects varied by food category, with the strongest improvements observed for breakfast cereals and biscuits.

**Table 4:** Relationship between FoPL type and ability to correctly rank products according to nutritional quality (*N* = 1,500).

Product choice	OR	95% CI	*p*-value
Yogurt			
Health Warning	0.96	1.03–2.01	0.031^*^
Nutri-Score	1.00	0.71–1.40	0.988
HSR	1.44	0.68–1.35	0.811
MTL	1.43	1.03–2.00	0.033^*^
Biscuits			
Health Warning	0.24	0.16–0.35	<0.001^*^
Nutri-Score	0.35	0.24–0.51	<0.001^*^
HSR	0.35	0.26–0.58	<0.001^*^
MTL	0.38	0.26–0.56	<0.001^*^
Breakfast cereals			
Health Warning	1.18	0.83–1.67	0.366
Nutri-Score	2.19	1.55–3.11	<0.001^*^
HSR	2.16	1.52–3.09	<0.001^*^
MTL	2.95	2.09–4.18	<0.001^*^
All products			
Health Warning	1.83	1.37–2.44	<0.001^*^
Nutri-Score	1.56	1.16–2.10	0.003^*^
HSR	1.46	1.78–1.98	0.014^*^
MTL	2.18	1.63–2.91	<0.001^*^

^*^The RI label served as the reference category in the multivariate ordinal logistic regression analyses. Models were adjusted for age, sex, place of residence, marital status, education level, occupation, household, and grocery shopping responsibility. OR, odds ratio; CI, confidence interval; ^*^p < 0.05.

**Figure 5:**
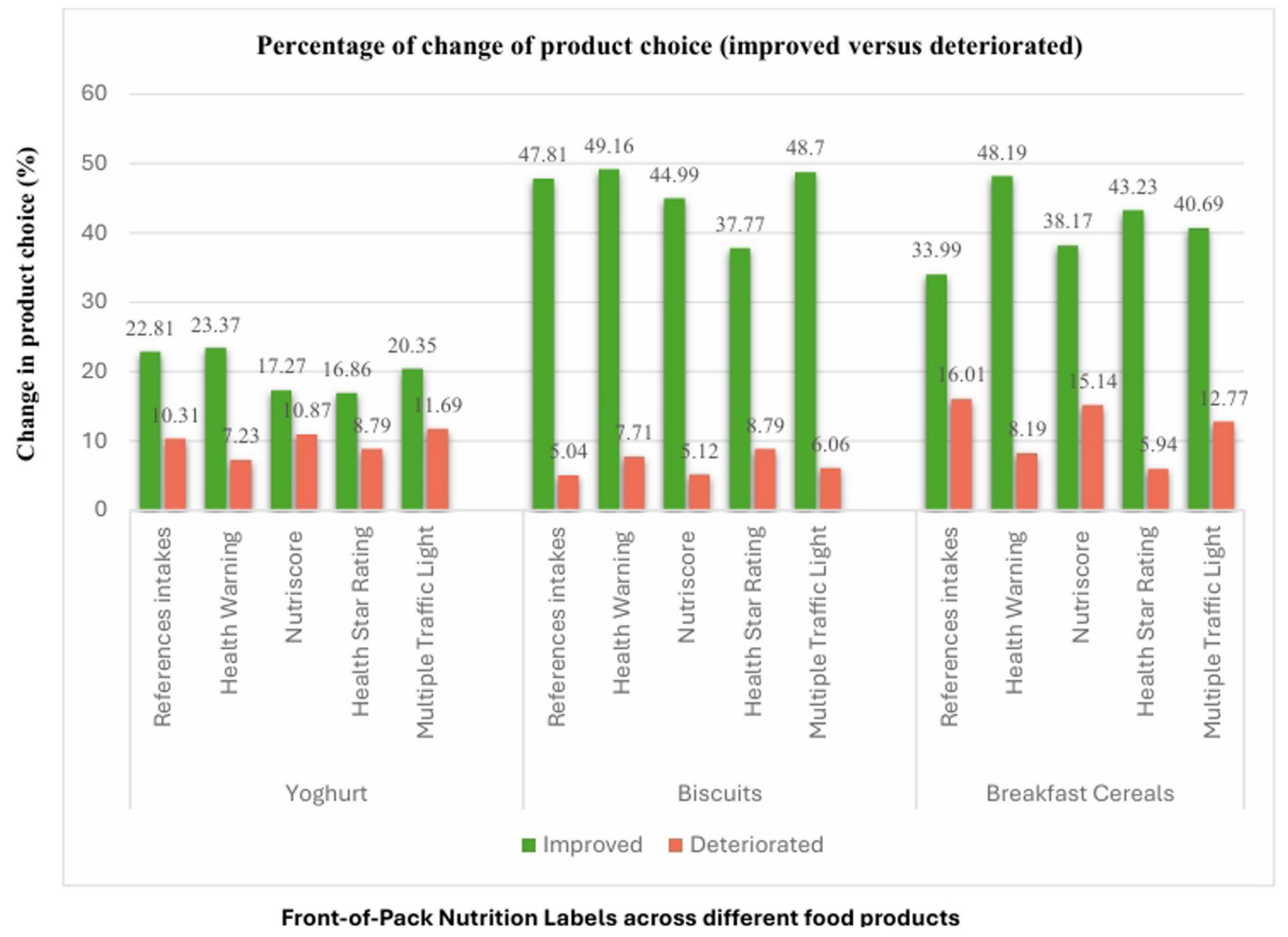
Change in product choice with and without nutrition labels for yoghurt, biscuits, and breakfast cereals.

As shown in Figure 5, across all label types, the proportion of participants who improved their selections—by choosing products with better nutritional quality profiles—was greater than the proportion whose choices deteriorated. The degree of improvement varied by product category. Biscuits showed the highest improvement, with the RI (49.04%) and Health Warning (48.99%) labels being the most effective. Nutri-Score also led to substantial improvement (41.72%). Deterioration rates for biscuits remained low across all labels, with the highest at 9.79% for HSR.

Breakfast cereals exhibited the most notable improvement with the Health Warning label (46.62%), followed by MTL (38.74%) and HSR (37.41%). However, deterioration rates were also higher in this category, reaching 17.2% under the RI label and 16.56% under Nutri-score. In contrast, yoghurt showed the smallest overall change. Improvements ranged from 18.21% (Nutri-Score) to 23.89% (RI), whereas deterioration ranged from 6.42% (Health Warning) to 12.25% (MTL).

Table 5 presents the association between sociodemographic characteristics and changes in nutritional quality across yogurt, biscuits, and breakfast cereals. Significant associations were observed for gender in biscuits and breakfast cereals (*p* = 0.009 and *p* = 0.020, respectively), place of residence in biscuits and breakfast cereals (*p* = 0.019 and *p* < 0.001), responsible for grocery shopping in yogurt (*p* = 0.012), and self-reported nutrition knowledge in biscuits (*p* = 0.004).

**Table 5:** Association between sociodemographic characteristics and changes in nutrition quality of the three food products (*N* = 1,500).

Variable	Yogurt		Biscuits		Breakfast cereals	
	Improved	Deteriorated	Improved	Deteriorated	Improved	Deteriorated
	*N* = 313	*N* = 142	*N* = 682	*N* = 104	*N* = 558	*N* = 190
Age						
<28	127 (40.58)	50 (35.21)	234 (34.31)	30 (28.85)	171 (30.65)	71 (37.37)
28–40	115 (36.74)	47 (33.10)	256 (37.54)	36 (34.62)	206 (36.92)	74 (38.95)
≥40	71 (22.68)	45 (31.69)	192 (28.15)	38 (36.54)	181 (32.44)	45 (23.68)
*p* value	0.123		0.204		0.057	
Gender						
Male	135 (43.13)	63 (44.37)	290 (42.52)	59 (56.73)	206 (36.92)	89 (46.84)
Female	178 (56.87)	79 (55.63)	392 (57.48)	45 (43.27)	352 (63.08)	101 (53.16)
*p* value	0.885		0.009*		0.020*	
Place of residence						
Central	63 (20.13)	26 (18.31)	157 (23.02)	12 (11.54)	124 (22.22)	16 (8.42)
Eastern	91 (29.07)	30 (21.13)	195 (28.59)	31 (29.81)	161 (28.85)	49 (25.79)
Northern	57 (18.21)	29 (20.42)	118 (17.30)	23 (22.12)	89 (15.95)	55 (28.95)
Southern	34 (10.86)	23 (16.20)	87 (12.76)	22 (21.15)	75 (13.44)	33 (17.37)
Western	68 (21.73)	34 (23.94)	125 (18.33)	16 (15.38)	109 (19.53)	37 (19.47)
*p* value	0.270		0.019*		<0.001*	
Marital status						
Unmarried	174 (55.59)	71 (50.00)	339 (49.71)	44 (42.31)	260 (46.59)	88 (46.32)
Married	139 (44.41)	71 (50.00)	343 (50.29)	60 (57.69)	298 (53.41)	102 (53.68)
*p* value	0.314		0.191		0.999	
Educational level						
High school or less	50 (15.97)	27 (19.01)	99 (14.52)	19 (18.27)	94 (16.85)	28 (14.74)
Diploma/University	196 (62.62)	90 (63.38)	440 (64.52)	64 (61.54)	336 (60.22)	131 (68.95)
Postgraduate	67 (21.41)	25 (17.61)	143 (20.97)	21 (20.19)	128 (22.94)	31 (16.32)
*p* value	0.538		0.607		0.080	
Employment status						
Employed	194 (61.98)	91 (64.08)	426 (62.46)	72 (69.23)	333 (59.68)	120 (63.16)
Unemployed & Student	119 (38.02)	51 (35.92)	256 (37.54)	32 (30.77)	225 (40.32)	70 (36.84)
*p* value	0.745		0.221		0.446	
Monthly income level						
<7,750	112 (35.78)	41 (28.87)	200 (29.33)	36 (34.62)	168 (30.11)	56 (29.47)
7,750–15,300	99 (31.63)	61 (42.96)	258 (37.83)	39 (37.50)	195 (34.95)	79 (41.58)
>15,300	102 (32.59)	39 (27.46)	224 (32.84)	28 (26.92)	194 (34.77)	55 (28.95)
*p* value	0.055		0.400		0.206	
Responsible for grocery shopping						
Yes	132 (42.17)	76 (53.52)	321 (47.07)	52 (50.00)	260 (46.59)	89 (46.84)
No	78 (24.92)	38 (26.76)	168 (24.63)	23 (22.12)	142 (25.45)	51 (26.84)
All family members	103 (32.91)	28 (19.72)	192 (28.15)	29 (27.88)	156 (27.96)	50 (26.32)
*p* value	0.012*		0.818		0.884	
Perception’ participant of their diet						
Very well balanced	27 (8.63)	14 (9.86)	60 (8.80)	13 (12.50)	44 (7.89)	18 (9.47)
Balanced	122 (8.63)	42 (29.58)	265 (38.86)	32 (30.77)	212 (37.99)	74 (38.95)
Somewhat balanced	120 (38.34)	67 (47.18)	281 (41.20)	44 (42.31)	243 (43.55)	74 (38.95)
Unbalanced	44 (14.06)	18 (12.68)	76 (11.14)	14 (13.46)	58 (10.39)	24 (12.63)
*p* value	0.207		0.339		0.611	
Nutritional knowledge						
Extensive knowledge	116 (37.06)	45 (31.69)	253 (37.10)	40 (38.46)	214 (38.35)	65 (34.21)
Sufficient knowledge	142 (45.37)	75 (52.82)	344 (50.44)	38 (36.54)	270 (48.39)	101 (53.16)
Limited knowledge	48 (15.34)	19 (13.38)	69 (10.12)	22 (21.15)	65 (11.65)	17 (8.95)
No knowledge	7 (2.24)	3 (2.11)	16 (2.35)	4 (3.85)	9 (1.61)	7 (3.68)
*p* value	0.541		0.004*		0.170	

Data are presented as numbers *n* (%). *p* value was calculated by chi-squared/Fisher–Freeman–Halton tests, ^*^p < 0.05.

## 4 Discussion

In this study, we examined the effectiveness of five different FoPL systems in encouraging healthier dietary decisions and improving objective understanding among a demographically diverse Saudi population with high self-reported nutritional awareness, using an online questionnaire. Among the labels tested, the MTL label was the most effective across all food categories, leading to an average improvement of 31% in correct product selection, followed by Health Warnings (28%), Nutri-Score (27%), HSR (24%), and RI (12%). The MTL was also significantly associated with more accurate rankings of nutritional quality (OR = 2.18, *p* < 0.001), particularly for breakfast cereals and biscuits. These findings are particularly relevant in the Saudi context, where FoPL policies remain largely voluntary (4), highlighting the potential role of effective label formats in supporting national nutrition strategies.

Label effectiveness varied by product type, with the greatest impact observed for biscuits and breakfast cereals. This aligns with trends in the Saudi market, where baked goods account for approximately 26% of packaged food sales (22). Notably, none of the tested FoPLs significantly influenced participants’ choices for yoghurt, possibly reflecting the widespread perception of yoghurt as inherently healthy and less in need of evaluation through labelling cues (23). In addition, variations in FoPL effectiveness across gender, place of residence, shopping responsibility, and nutrition knowledge suggest that consumer characteristics influence how labels are interpreted and used.

The findings correspond with prior research (24, 25) showing that FoPLs can facilitate healthier food choices. Studies conducted in Riyadh and Al-Hasa reported adequate consumer awareness and understanding of food labelling, which may have contributed to the overall effectiveness of FoPLs observed in this study. Similarly, Shin et al. demonstrated that both warning labels and nutrition scores improved food and beverage selections compared with no labelling (26). These findings reinforce the role of FoPLs as practical tools for supporting healthier consumer choices.

In terms of nutritional quality, Nutri-Score was particularly effective for biscuits compared with RI, aligning with the results of Egnell et al. (11), who reported that Nutri-Score consistently outperformed other labelling systems across various food categories. Talati et al. (27) also observed that HSR increased consumers’ willingness to pay for healthier options, especially indulgent items such as cookies. For breakfast cereals, our findings showed significant associations with Health Warnings (OR = 0.56, *p* < 0.001), HSR (OR = 0.61, *p* = 0.002), and MTL (OR = 0.70, *p* = 0.021). These relatively lower ORs suggest modest improvements in food choice, potentially due to complex health perceptions associated with cereals. This aligns with the observations of Talati et al. (27), who found that consumers were willing to pay significantly more for healthier cereal variants under HSR conditions—ranging from $0.74 for cookies to $1.94 for cornflakes—highlighting the strong utility of HSR.

Interestingly, Nutri-Score did not significantly influence the participants’ choice of cereals (OR = 0.90, *p* = 0.501). However, a study in Belgium found that cereals with higher Nutri-Score ratings (A or B) were more likely to display health claims and had undergone reformulation, such as reductions in sodium (−20%) and sugar (−5%) and increases in fibre (+3%) and protein (+2%) (27). These results suggest that while Nutri-Score may not always drive immediate behavioral changes, it can promote industry reformulation and enhance the nutritional quality of products (28). Such reformulation is encouraged through national guidelines in Saudi Arabia, with FoPLs potentially supporting these efforts.

International literature frequently highlights Nutri-Score as the most effective FoPL for encouraging healthier choices across diverse populations (11–13); however, the current findings indicate that MTL yielded the greatest improvements in both product selection and nutritional ranking across all tested food categories. A possible explanation is MTL’s intuitive, colour-coded format, which may align more closely with Saudi consumers’ visual preferences and cognition for direct and easily interpretable information. The traffic-light scheme—highlighting nutrient-specific values for fat, sugar, and salt—facilitates rapid evaluation and enhances label comprehension, particularly among consumers with limited nutritional knowledge or prior exposure to label formats (11, 18). In contrast, Nutri-Score uses a summary letter grade (from A to E) to show the overall health of a product, which, although scientifically developed, is based on a scoring system that combines several nutritional factors (10) that may require greater familiarity or background knowledge to interpret effectively, especially in populations with limited exposure (20).

This difference in interpretability is consistent with our perception data, which showed that over half of the participants found MTL the easiest to understand and preferred it over other formats, including Nutri-Score. These findings align with existing evidence that colour-coded FoPLs outperform summary labels in unfamiliar settings, especially among consumers with low nutritional literacy or limited prior exposure (8, 13, 25).

Previous research supports these findings. Talati et al. (18) noted that in non-European contexts, nutrient-specific FoPL systems such as MTL are often perceived as more accessible than summary labels. Similarly, Aguenaou et al. (20) found that Moroccan consumers—culturally and regionally similar to Saudi consumers—had lower familiarity with Nutri-Score, limiting their ability to use it effectively. Local studies by Al-Barqi et al. (8) and Binobead et al. (25) further support this, revealing that Saudi consumers often lack the nutritional literacy needed to interpret complex labels and therefore prefer visually simple, intuitive systems such as MTL.

Moreover, regional policy differences may influence label familiarity and effectiveness. In Saudi Arabia and the United Arab Emirates, MTL labelling was introduced voluntarily between 2018 and 2019 (25), increasing public exposure. In contrast, Nutri-Score has not been officially endorsed by the Saudi Food and Drug Authority and remains uncommon on packaging in local markets (26). The lack of public education campaigns and regulatory implementation likely contributed to the lower perceived clarity and preference for Nutri-Score observed in this study. Therefore, despite strong international support for Nutri-Score, the present findings suggest that MTL may currently be more appropriate and impactful in the Saudi context. This highlights a gap between international evidence and national regulatory adoption, suggesting that policy decisions in Saudi Arabia should consider both global best practice and local consumer understanding when selecting a unified FoPL system.

The FoPL system also has broader implications for public health policy at the industry level. Evidence suggests that FoPL implementation can encourage food manufacturers to reformulate products by reducing levels of sugar, sodium, and unhealthy fats, thereby improving the overall nutritional quality of the food supply (29). Findings from countries implementing warning label systems support these effects, showing consistent improvement (29). Overall, FoPL extends beyond influencing consumer choices by also contributing to a healthier food environment through changes in food production practices.

A key strength of the current study is the use of a quota-based sampling strategy, which enhanced the representativeness of the sample and supports the generalisability of the findings. Nonetheless, a few limitations should be noted. The cross-sectional design and reliance on a self-administered online questionnaire introduce potential biases such as self-reporting bias and underrepresentation of specific demographic groups, including older adults and those with lower levels of education. Future research should further assess FoPL effectiveness under real-world conditions and potential regulatory developments in Saudi Arabia.

## 5 Conclusion

FoPL systems are essential tools for guiding consumers toward healthier food choices. This study offers valuable insights into the comparative effectiveness of different FoPL formats among Saudi consumers. Overall, interpretive FoPL systems were associated with greater support and stronger indications of healthier food choice guidance than non-interpretive or numeric labelling formats. Among the labelling systems assessed, MTL, Health Warning symbols, and Nutri-Score were associated with higher levels of participant support. In particular, MTL demonstrated potential to facilitate healthier food choices, likely due to its simplicity and clarity. Nutri-Score also appeared promising; however, its performance may be constrained in the absence of targeted consumer education, particularly among individuals from lower socioeconomic backgrounds.

Overall, these findings underscore the importance of considering cultural context and consumer understanding when evaluating FoPL format. In Saudi Arabia, where FoPL implementation remains largely voluntary under the Saudi Food and Drug Authority, the results support consideration of a standardized, interpretive FoPL approach, such as MTL, accompanied by targeted consumer education to enhance its impact. Such measures may contribute to national efforts to improve dietary behaviors and reduce diet-related health risks. In addition, combining FoPL adoption with education campaigns and sustainable dietary practices may enhance behavioral change and support long-term improvements in population health.

## Data Availability

The original contributions presented in the study are included in the article/supplementary material, further inquiries can be directed to the corresponding author.
